# An integrative review and practical guide to team development interventions for translational science teams: One size does not fit all

**DOI:** 10.1017/cts.2021.832

**Published:** 2021-08-09

**Authors:** Sydney R. Begerowski, Allison M. Traylor, Marissa L. Shuffler, Eduardo Salas

**Affiliations:** 1 Clemson University, Clemson, SC, USA; 2 Rice University, Houston, TX, USA

**Keywords:** Translational science, science of team science, team effectiveness, team behavior, team development interventions

## Abstract

As the need to tackle complex clinical and societal problems rises, researchers are increasingly taking on a translational approach. This approach, which seeks to integrate theories, methodologies, and frameworks from various disciplines across a team of researchers, places emphasis on translation of findings in order to offer practical solutions to real-world problems. While translational research leads to a number of positive outcomes, there are also a multitude of barriers to conducting effective team science, such as effective coordination and communication across the organizational, disciplinary, and even geographic boundaries of science teams. Given these barriers to success, there is a significant need to establish team interventions that increase science team effectiveness as translational research becomes the new face of science. This review is intended to provide translational scientists with an understanding of barriers to effective team science and equip them with the necessary tools to overcome such barriers. We provide an overview of translational science teams, discuss barriers to science team effectiveness, demonstrate the lacking state of current interventions, and present recommendations for improving interventions in science teams by applying best practices from the teams and groups literature across the four phases of transdisciplinary research.

## Introduction

Interdisciplinary collaborations are rising in frequency as researchers strive to tackle complex clinical and societal problems. These teams, known as translational science teams, combine perspectives across disciplines and place emphasis on translating findings from clinical environments to the larger society. Translational research has led to numerous positive outcomes, including breakthrough findings, increased citation counts, and substantial advancements in clinical research [1,2]. Specifically, novel combinations of disciplines have led to science teams with substantial levels of creativity and innovation, leading to the production of higher impact science [3,4]. In clinical environments, a translational approach to research has led to significant advancements in many fields including, but not limited to, orthopedics, cancer care, and pediatrics [5–7].

The shift toward heightened collaboration that spans disciplinary, organizational, and even geographic boundaries suggests a fundamental transformation in the production of high-impact science [8]. Notably, this shift sparked the creation of an emerging field that investigates various factors affecting cross-disciplinary (CD) science teams and their effectiveness. This field, more formally known as the Science of Team Science (SciTS), seeks to apply fundamental knowledge of teams in the context of CD and translational research. In addition to the many benefits of CD teams, SciTS researchers have also noted critical barriers to science team success [6,9].

Despite identifying such barriers, there is little in the literature that presents practical solutions to enhance translational science team effectiveness. What literature does exist tends to emphasize training interventions that require extensive time and monetary resources for teams. Though the team science literature has captured multiple easily implementable solutions [10], many of these interventions have not received attention in the context of translational science. Moreover, scientists interested in implementing these best practice interventions may not have relevant information on how to do so. Nonetheless, translational research teams are on the rise, resulting in a significant need to develop practical team interventions that address common barriers to success. In doing so, emerging translational scientists will be able to implement interventions to enhance team effectiveness and therefore achieve the numerous benefits of CD teams as translational approaches become the new face of science.

To address this gap, the purpose of this review is fourfold: 1) to provide an overview of translational science teams; 2) to discuss barriers to science team effectiveness; 3) to demonstrate the lacking state of current interventions; and 4) to present recommendations for improving interventions in translational science teams by applying best practices from the teams and groups literature. More specifically, our review begins with an overview of key elements that define translational science teams, their distinction from other teams, and the incremental value of translational teamwork in knowledge production and research translation. Next, we review barriers to effective collaboration from both an overall perspective and a team’s perspective. We then review key takeaways from the current state of science team interventions, addressing both their potential value in enhancing translational science as well as their limitations in functionality on a team level. Finally, using Hall and colleagues’ pivotal work detailing the four phases of CD research [11], we integrate these literatures by demonstrating applications of team development interventions and key facilitators of effectiveness across each phase and contribute practical steps for implementation. With this integrative review, we hope to offer translational scientists a richer understanding of team science barriers while also equipping them with necessary tools to overcome such obstacles, in turn enhancing future team science success.

### Translational Science Teams

The prevalence of science teams and CD research has risen drastically as science continues to demand more novel findings and unique solutions to increasingly complex problems. [12,13] In general, science teams represent groups of individuals that work interdependently to achieve intellectual outputs (e.g., knowledge creation, knowledge integration). Science teams vary significantly in structure from that of more traditional teams (e.g., corporate, manufacturing, military) in that they have greater emphasis on knowledge production, have more permeable boundaries that reflect the changes in goals over time, and are highly variable in team size [14]. These differences underscore critical team processes (e.g., coordination, communication) and approaches to research (e.g., multidisciplinary, transdisciplinary [TD]) needed to realize the benefits of team science.

Translational science teams hold these characteristics but place unique emphasis on translating findings into clinical practice from both micro and macro perspectives. Translational scientists work to bridge knowledge from various disciplines to achieve novel findings, seek to advance research findings into clinical practice, disseminate clinical practice into communities, and ultimately improve overall global wellness [15]. In fact, the emergence of clinical and translational science is stated to be a key paradigm in optimizing the intersection of scientific discovery and healthcare delivery [16].

These intricate, overarching goals require translational teams to have complex compositional attributes. Translational research teams typically include scholarly roles (e.g., primary investigators, graduate students) in addition to practitioner roles across various professions [11]. Science teams must balance compositional factors such as diversity in disciplinary backgrounds, geographical location, and cultural and organizational norms and values. While these factors can create challenges, it is the complexities behind science team composition that create value in the translational approach [1]. This diversity in representation is the key not only for successful integration of disciplinary knowledge but also for translation back into practice.

Given the emphasis on integrating knowledge and practice, translational science teams are increasingly using a TD approach to better research and manage the prevention, diagnosis, and treatment of complex diseases in order to tackle both the clinical and societal implications of such diseases. For example, diseases such as obesity, smoking, and Alzheimer’s are complex biosocial–environmental problems that require the integration of various disciplines to effectively address both the micro (e.g., cellular) and macro (e.g., societal) nuances of the diseases [17]. A TD approach to research is inherently more complex than other forms of CD research as it transcends beyond simple integration of multiple disciplines [18]. In particular, the end goal of TD research is to yield innovative solutions to a specific clinical, scientific, or societal problem that can be implemented into practice, placing emphasis on the translation of research findings into practical solutions [19]. This is often accomplished through intensive, integrative processes that synthesize discipline-specific theories, methods, and translational strategies into new conceptual frameworks and theories that extend beyond their disciplinary origin [19]. The TD research process can be either discovery-oriented or use-oriented; however, the intricacies of integration to achieve novel scientific discoveries can be time-intensive, spanning multiple years as replication studies are conducted to validate findings prior to translation into practice. Given the potential benefits of TD research, translational science teams are continuing to rise in frequency. Nonetheless, the complexities underlying effective TD research cannot be overlooked.

### Barriers to Effective Translational Team Science

Translational science teams face a myriad of challenges that span individual, team, and organizational levels, many of which stem from the integration and implementation of a TD approach. From a larger organizational view, TD research efforts can be complicated by a lack of institutional support [20,21]. More specifically, lack of institutional awareness in both the value of TD research as well as TD research operations can lead to limited support in terms of financial resources and infrastructure (e.g., technological resources) [22]. In addition, TD research often requires higher levels of financial support; however, institutions can be hesitant to invest in new TD projects due to the highly complex goals associated with TD research as well as initial lags periods of TD research that masks themselves as low productivity [11,23]. Even so, there is a growing body of literature that suggests emphasis on collaborative research by academic partners through promotion and tenure policies impacts collaborative knowledge production [24]. Moreover, a number of collaboration centers (e.g., Clinical and Translational Science Award Institutes, university collaboration centers) dedicated to fostering and maintaining CD approaches to research are emerging to address these organizational concerns [25].

On an individual level, knowledge, skills, and abilities (KSAs) can influence team science effectiveness. In terms of knowledge, many researchers are trained in a discipline-specific manner, not only limiting knowledge but also an understanding of research best practices (e.g., methodologies, translational strategies) to a few domains [26]. Team scientists should also demonstrate competencies unique to collaborative research, including interdisciplinary skills, reflective behavior, and recognizing disciplinary perspectives [27]. Whereas interdisciplinary skills refer to the ability to apply various disciplinary perspectives to make connections across disciplines, reflective behavior focuses on the ability to recognize general approaches to problem-solving and occasional need for reconsideration. Finally, team scientists should recognize disciplinary perspectives such that they understand how to apply various content, methods, and boundaries of different disciplines upon situational needs. Indeed, individual competencies serve as key leverage points for influencing team processes and effectiveness [28,29]. Without the development of these interdisciplinary competencies, translational scientists will lack the KSAs to effectively engage with team members in the knowledge production process. For translational scientists interested in further refining these competencies, there are existing field guides (e.g., the NCI Collaboration and Team Science Field Guide) that address the development of individual KSAs in the context of CD research [30,31].

In addition to these barriers, there are also several team-level challenges inhibiting translational science team effectiveness. First, limited guidance on TD best practices poses significant difficulties for translational teams. [6] When team members represent a wide variety of disciplinary and institutional backgrounds, team processes like communication, coordination, and connection become increasingly difficult [23,32]. Similarly, there are inherent challenges in integrating multiple disciplinary theories, models, and methodological practices. Simply understanding how various fields complement one another requires additional time and skills, such as learning discipline-specific jargon and engaging in unfamiliar environments [33]. Finally, TD research is often extensive given the scope of problems translational scientists are seeking to solve. When spanning organizations, disciplines, and often geographic boundaries, what are otherwise simple research project management practices now require complex coordination and maintenance. Time management, coordination across scientists and clinical investigators, and other administrative tasks (e.g., resource allocation) pose significant barriers if not executed correctly [6,32]. Many of these barriers are consistent with common challenges to effective teamwork [34], but are often exacerbated by the complexity of combining multiple disciplines, demonstrating a significant need to address these barriers to enhance translational team effectiveness.

### Current Interventions for Science Teams

To combat these common barriers, our review revealed several key facilitators to effective translational science, including establishing a shared vision, developing and maintaining clear expectations, cultivating team trust, and promoting effective communication. There is overwhelming support demonstrating the importance of a shared vision, whether it be in terms of the team’s mission, research goals, coordination efforts, or even plans for publications [24,35–38]. Similarly, developing and maintaining clear expectations throughout the TD research process aid team effectiveness [35]. In particular, management of expectations regarding roles, group norms, and other team phenomena can influence the development of trust [37]. Trust is especially critical when translational teams begin to span geographical boundaries, often requiring some virtual elements to maintain communication [9]. Promoting and modeling effective communication is also key to facilitating team effectiveness, whether it be across geographic boundaries or simple day-to-day best practices [9,35]. Within communication practices, promoting disagreements while simultaneously controlling conflicts can help maintain shared visions while also encouraging the development of innovative ideas [37]. Finally, strategically identifying team members, purposefully building the team, and assessing collaboration readiness among team members are crucial facilitators of effectiveness when beginning the translational research process [24,37,38].

Despite the identification of facilitators, practically implementable interventions for enhancing translational science team effectiveness remain limited. Most interventions for science teams are multiday workshops that address the development of individual competencies for TD research [39,40]. These workshops tend to focus on fostering the KSAs needed to approach TD research [26,41]. While many render successful results, such workshops are demanding in time and resources. Though fewer in number, there are also interventions focused on competency development through mentorship programs [24,40]. However, these programs tend to target graduate students and post doctorates as opposed to more senior scientists [42]. Thus, consistent with calls previously expressed in the literature [2,42], there is still a significant need for practical, team-orientated interventions that can enhance facilitators of translational research and address common barriers to TD effectiveness.

### Applying Team Interventions to Translational Science Teams

Team science researchers have spent decades investigating the effectiveness of team development interventions (TDI) across a wide range of settings. TDIs describe “actions taken to alter the performance trajectories of teams,” and can range from intensive, months-long interventions to shorter-term exercises [43]. Table 1 provides an overview of the TDIs discussed in this article. While we provide guidance for implementing TDIs in the coming sections, Table 1 also provides outside resources for designing and implementing each of the interventions discussed in our paper.

**Table 1.** Types of team development interventions and resources for implementation

Team development intervention	Definition	Resources for implementation
Team training	A set of strategies or instructional processes based on the science and practice of designing and delivering instruction to ensure understanding and enactment of appropriate team competencies	King et al.^47^ Salas & Cannon-Bowers^48^ Salas et al.^49^
Team charters	Codified plans for how a team will manage teamwork activities	Mathieu & Rapp^50^ Norton & Sussman^51^
Team coaching and leadership	A direct interaction with a team intended to help members make coordinated and task-appropriate use of their collective resources in accomplishing teamwork	Day et al. ^52^ Hackman & Wageman^53^ Traylor et al. ^54^
Team huddles and debriefing	A type of team meeting in which people a type of work meeting in which people discuss, interpret, and learn from a recent event during which they collaborated	Allen et al.^55^ Flanagan^56^ Keiser & Arthur^57^ Reyes et al. ^58^ Smith-Jentsch et al.^59^

Notably, team scientists differentiate between two types of TDIs: *training interventions* and *process interventions* [43]. Our paper focuses on team process interventions, which place emphasis on improving team members relationships or how teams work together. Process-focused TDIs tend to be shorter term and more easily implemented but still effective in improving team outcomes throughout a team’s lifespan [43]. However, many of the interventions we describe can be enhanced when teams have a rudimentary understanding of team functioning which can be established via team training. Indeed, because team training often teaches teams how to engage in the types of process-based interventions discussed in this study, it is extremely effective in improving team performance [44,45]. Although a more detailed discussion of team training is beyond the scope of this study, Table 1 provides additional resources for designing and implementing team training programs.

Importantly, TDIs are not a “one size fits all” solution, and instead should be targeted toward a team’s current challenges or their research phase [10]. TD teamwork is typically conceptualized in terms of four phases: development, conceptualization, implementation, and translation. It is important to note that there are other methods for classifying the current state of a science team. For example, the current stage of a team can be conceptualized in terms of general classification (e.g., formative, intermediate, concluding), productivity assessments (e.g., publication counts, levels of impact), or even relative stage to the team’s half-life [11,46]. The way in which the team’s development is operationally defined can significantly impact the type of intervention implemented to enhance productivity. Given that most translational teams go through the four phases as defined by Hall and colleagues, we use these phases to guide our review, providing an overview of each phase of research, relevant team competencies, and suggestions for interventions. Table 2 provides a summary overview of these interventions and includes guiding questions in implementing each intervention.

**Table 2.** Guiding questions for team interventions by TD research phase and relevant team constructs

TD research phase	Relevant team constructs	Team development interventions	Guiding questions
Development	Shared mental models, role clarity, psychological safety	Team charters	- Has the team established a set of shared goals? (SMM) - Has the team established members’ relevant expertise? (role clarity) - Have we considered the perspective of members from all disciplines? (psychological safety)
Conceptualization	Shared mental models, team conflict, active listening, psychological safety	Team huddles, team coaching, team leadership	- Are ideas and insights from all teammates being considered? (psychological safety) - Are all team members on the same page about the problem and best approaches for solving it? (SMMs) - Are team members engaging in respectful disagreement and discourse as they generate solutions? (conflict, active listening)
Implementation	Shared mental models, coordination, transactive memory systems	Team huddles	- Do team members have a shared understanding of their and others’ roles on the team? (SMM) - Is the team effectively integrating their knowledge to generate a solution? (TMS, coordination)
Translation	Shared mental models, transactive memory systems	Team debriefing	- Has the team established shared goals for translation? (SMM) - Does the team have a shared sense of how their activities support the team’s goals for translating findings into their context? (SMM) - Does the team effectively integrate their knowledge as they pursue translational goals? (TMS)

*Note.* TD, transdisciplinary; SMM, shared mental model; TMS, transactive memory system

The current review focuses on TDIs that are well-established in the team science literature [10,43] and relevant when considering the specific nature of translational science teams. As such, the following sections focus on identifying key constructs and team development interventions broken down by the translational research stage.

#### Development

The first phase of TD teamwork is the *development phase*, in which teams are expected to define the scientific or societal problem space of interest [11]. For example, teams may identify concepts that fall within the problem space and establish the boundaries of the problem the team will address. The development phase requires teams to effectively share ideas and communicate their expertise, begin to generate creative ways of looking at the problem space, and set expectations about the problems at hand and the role of each collaborator.

The development phase captures a team transition, or a time period when teams are required to define and set goals and strategize about how to tackle a problem [60]. Research demonstrates intellectual teams (versus teams focused on physical tasks) tend to place more importance on transition processes and that intellectual teams rely on transition processes more than action processes, which are focused on task execution [61].

For teams at inception, a team charter can be an effective intervention for guiding goal-setting discussions and crystallizing a strategy for addressing the team’s goals. Team charters describe an intervention used to develop team norms and processes and define various aspects of teamwork, including purpose and mission statements, operating guidelines, behavioral norms, and performance management practices [10]. Although the exact contents of a team charter may vary from team to team, the initial meeting to form a team charter can also provide a guided discussion format for teams to define their problem space, as is necessary for the development phase [11]. Despite widespread use in practice, research on the use of team charters is somewhat scarce. Studies have focused primarily on student project teams; however, in student teams, team charters are linked with higher levels of satisfaction and performance [10].

Team charters provide a structured way for teams to develop a shared mental model (SMM) early in the project, setting the team up for success down the road [50]. A SMM describes a team’s common, overlapping understanding of task requirements, procedures, and role responsibilities [48]. SMMs have demonstrated links with high team performance across a variety of contexts. [62] If teams develop a charter early on, they are also better able to manage disruptions because they possess guidelines for behavior in contingency situations [63].

Team charters are particularly important for translational teams. They provide an opportunity for guided discussion clarifying team members’ areas of expertise and relevant knowledge, and to get everyone on the same page as they begin a project. In translational teams, members may have a limited understanding of team members’ areas of expertise or may have very different perspectives on the project. Developing a team charter can be an opportunity to develop a shared vision and vocabulary to ensure team members are on the same page about the problem space and tools available to solve the problems at hand. Without a team charter, a translational team may have more difficulty integrating disciplines as well as addressing complexities and conflicts as they arise.

Although the specific content of a team’s charter might vary based on project needs, in general, developing a team charter should involve discussing and documenting the team’s purpose and mission statement, establishing guidelines for communication and norms for behavior, and outlining performance management practices. For example, teams may discuss their project’s purpose, whether and what the team’s specific goals are, and what the team’s timeline for achieving their goals are. To establish communication guidelines and norms, teams may discuss how often they will meet or what the best mode of communicating with team members between meetings will be. Finally, to clarify performance expectations, the team might discuss appropriate avenues for providing feedback and performance issues within the team.

#### Conceptualization

The conceptualization phase involves developing novel research questions, hypotheses, a conceptual framework, and a research design that integrates collaborators’ disciplinary perspectives and knowledge domains to address the target problem in innovative ways [11]. This phase occurs after teams have formed and established overarching goals and a problem space. Ideally, team members will feel free to share their unique perspectives, give critical feedback, and debate ideas. Effective processing during conceptualization helps ensure teams are generating creative solutions to problems and effectively integrating members’ diverse perspectives.

Team leadership and coaching are TDIs that are particularly important for guiding teams in the conceptualization phase and helping create an environment where members can share and debate ideas freely. Team scientists think about two types of team leadership interventions—while some interventions may be focused on how to develop team leaders, a second set of approaches is focused on how team leaders can intervene to help their team [10]. Team leadership training typically involves a time-intensive, multisession effort to build the KSAs necessary to become an effective team leader [43].

In contrast, team coaching can be thought of broadly as a particular set of behaviors enacted by the team’s leader to help develop team processes or more narrowly as a specific, dedicated intervention conducted by an outside facilitator [54]. Although the literature on team coaching lacks systematic evaluation, reviews suggest that team coaching tends to be effective in improving team outcomes [10,54]. We focus here on team coaching interventions as a broader set of leader behaviors that can promote teamwork during the conceptualization phase. Regardless of format, effective team coaching is typically tailored to a team’s phase of development [53]. For example, coaches may focus on motivating team members as they begin working together or provide feedback or opportunities for team learning after a team is finished working together.

Effective team leadership and coaching can help teams maximize psychological safety and minimize team conflict—two constructs that are particularly pertinent for team creativity and information sharing [64,65]. Team psychological safety describes the shared sense that a team is safe for interpersonal risk taking including speaking up, debating ideas, or disagreeing with the team leader [66]. In translational teams, psychological safety is achieved if all team members, regardless of discipline, feel safe sharing their ideas. Moreover, it is especially important that translational team members feel safe engaging in intellectual debate about their goals, the best ways to approach problems, and how to translate findings to stakeholder groups [67].

Psychological safety is important for several team outcomes but is particularly relevant for team innovation, creativity, and learning [64]. However, translational teams may have difficulty developing psychological safety if there is a perceived hierarchy between disciplines. For example, in medicine, researchers tend to find that physicians view teams as more psychologically safe than nurses [68]. In translational teams where members have very different backgrounds, leaders should be aware of subtle differences in how each discipline is perceived or under-representation of certain disciplines in the team. For example, in a team of mostly physicians from across various disciplines, an organizational psychologist might need an extra nudge to feel safe speaking up.

Leaders are critically important for developing and maintaining psychological safety in teams [64,66]. For instance, leaders who show humility and inclusiveness are more likely to develop a psychologically safe environment for their team members [69]. In translational teams, leaders might promote psychological safety by responding openly to team members’ ideas or by being open about their own ideas or prior mistakes as the team brainstorms solutions to problems. Leaders are also well positioned to help manage team conflict. Team scientists typically discuss three types of team conflict: relationship conflict, which concerns team members’ feelings toward each other; process conflict, concerning disagreements about how to complete tasks; and task conflict, which concerns disagreements about how to resolve the problems or tasks at hand [70]. In general, teams with high levels of task conflict and low levels of process and relationship conflict are the highest performing [71]. Teams with moderate levels of task conflict also tend to be most creative [65]. While relationship conflict and process conflict tend to be detrimental to team performance, healthy levels of task conflict are an indicator that a team is freely sharing and debating ideas. Teams that can do so will be able to generate more creative, innovative solutions.

While team members may be the source of team conflict, team leaders can engage in coaching behaviors that help mitigate team conflict [54]. For example, leaders may provide mediation between two team members struggling with relationship conflict. Leaders can also promote an open environment by demonstrating interest in ideas from across disciplines and modeling discussion that provides constructive criticism of ideas to develop the best approaches or solutions.

However, more effective than mitigating team conflict may be helping translational teams promote task conflict and reduce relationship and process conflict. While task conflict is beneficial to many teams, it is vital to the performance of translational teams, whose work revolves around the ability to innovate and generate creative solutions to problems [67]. During team meetings, leaders might promote task conflict by asking team members to share counter-perspectives or to provide critical feedback on each other’s ideas. However, the team leader should work with team members to mitigate relationship conflict so that task conflict is driven purely by intellectual debate rather than by interpersonal issues.

#### Implementation

In the implementation phase, translational teams launch, conduct, and refine the planned TD research [11]. Whereas the first two phases of TD teamwork are focused on planning, the implementation phase is focused on action. Effective teamwork in this phase requires effective coordination across disciplines and efforts to keep the team on track to achieve its goals. In addition, team members in this phase may shift their focus from team-based discussions and goals to their individual roles within the team. However, teams that can maintain a team-centric focus are more likely to experience higher levels of performance [72].

As a team is working together to achieve their goals, effective TDIs may include various forms of “huddles.” Huddles describe a frequent form of structured communication among team members to plan for daily tasks and roles and to review any barriers or facilitators of the day’s work [73,74]. Huddles are an opportunity to check in on goal progress and ensure team members have the support and resources they need. Although huddles are commonly implemented across industries, research on the effectiveness of huddles is most robust in healthcare settings. Studies of huddles in healthcare indicate that the intervention is effective in improving both work and team processes and improved clinical outcomes [75].

Whereas team charters help teams develop a SMM, team huddles help ensure that teams maintain their SMMs. Over time, SMMs tend to degrade, especially for teams facing acute stress in executing their work [76]. During the implementation phase, a translational team should have a shared understanding of their overarching team goal and of each team member’s role in achieving that goal. As a team encounters bumps in the road, it may become unclear who needs backup or who should be responsible for addressing new tasks or issues that arise. A team that has developed and puts continuous effort into developing a SMM through regular team huddles is positioned to discuss challenges as they arise and fluidly implement workarounds or reassign tasks to the team members best equipped to manage each task. Maintaining a SMM is necessary for implicitly coordinating efforts across disciplines. A small amount of effort put into regularly touching base about specific issues can go a long way in boosting team performance.

Team huddles need not be a long or intensive team meeting, but a team leader or assigned facilitator may be best for guiding the team through these check-ins. For example, a facilitator may hold weekly or biweekly quick check-ins to ask team members about their progress, remind team members of their goals, and ask team members for feedback on improving processes or tweaking strategy. Alternatively, a team leader may conduct daily huddles virtually, asking team members to quickly respond with what they are working on in each day and asking what type of support could help them accomplish their goals.

#### Translation

The final phase of TD teamwork is the translation phase, which is focused on applying research findings to advance progress along the discovery–development–delivery pathway to ultimately provide innovative solutions to real-world problems [11]. In this phase, teams are still working toward a final goal of translating findings to stakeholder groups, but this phase also provides an opportunity for team reflection and learning. The translation of research to stakeholder groups is a key factor that distinguishes translational research from other forms of TD research [77]. Accordingly, ensuring a team is prepared to crystallize their findings and disseminate work to stakeholder groups is key.

In order to achieve this goal, it is vital for translational teams to consider translation at all phases as other TDIs are implemented. For example, team charters are intended to define a team’s purpose and mission statements [10]. Translational team charters should include a discussion of goals related to translation, and in the conceptualization phase, teams should continue to consider how their work will eventually inform translation-related goals. Similarly, team huddles may be designed to orient team members toward translation. During regular huddles, team leaders may prompt members to think about how their findings or progress in the day-to-day will inform eventual translation.

As a project comes to a close, teams may be interested in crystallizing knowledge and learning via a debrief or similar reflective activity [55]. Debriefs are similar to the huddles conducted in the implementation phase but are more focused on reflecting on the project as a whole and determining ways to improve teamwork in the long term. A large body of research on TDIs demonstrates support for team debriefs in improving future performance and maximizing team learning. Indeed, a recent meta-analysis of team debriefing found that, across several industries and team types, team debriefing improved effectiveness by more than 25% [57]. In TD collaborations, debriefs can go a step further by sowing the seeds for future collaborative efforts. Research indicates that positive experiences with TD teamwork promote future TD efforts. Individuals who have collaborated in the past and report strong ties between team members are more likely to continue the collaboration, express intent for future collaborations, and collaborate on grant proposals [78–80].

Similar to huddles, debriefs may be conducted by team leaders or by an outside facilitator. The leader might begin a debrief by providing a space for team members to talk about what went well and what could have gone better. They may follow with a solicitation for specific ideas on how to improve in the future. Finally, the leader may provide their own thoughts or perspective. During these sessions, it is important that team members feel free to speak up, particularly about errors or things that could have gone better in the project, to promote team learning [66]. To promote future collaboration, leaders may include a “looking forward” component to the debrief by asking participants what ideas they have for future collaboration or what they have learned that will inform their next collaborations.

Team scientists have suggested several recommendations for promoting effectiveness in team debriefs [55,81]. For example, leaders should create a supportive learning environment for debriefing by promoting psychological safety. Debriefs should also be “diagnostic” rather than evaluative—instead of focusing on how the team performed, debriefs should address specific ways to improve team processing and team interactions. Finally, debriefs should be focused. Although there may be an opportunity to discuss a wide range of topics, the team leader or outside facilitator should choose a few of the most salient issues or those most likely to impact how the team approaches their next project.

### Evaluating Intervention Effectiveness

Although evidence for the effectiveness of TDIs on the whole is robust, as noted in prior sections, some interventions remain understudied, particularly in translational contexts. Accordingly, translational scientists may be interested in evaluating TDIs after implementation. Evaluating TDIs requires both identifying the competencies necessary for effectiveness in a given context and then selecting measures to capture those competencies.

Broadly, team effectiveness can be assessed by the quality and quantity of a team’s outcomes, the satisfaction of its members, or the viability of a team over time [28]. These criteria can be contextualized for translational teams in line with expectations of teams at each stage of research, as described above, or in line with a team’s specific context. In our previous section on TDIs, we describe several team constructs that have robust links with team performance across contexts, including SMMs, role clarity, conflict management, psychological safety, active listening, and team coordination. Translational scientists may elect to evaluate the effectiveness of TDIs by assessing the impact of the intervention on one or more of these constructs. Alternatively, a translational scientist may identify contextualized team performance outcomes that are aligned to the research team’s goals. Importantly, criteria for effectiveness should also align with the chosen TDIs, as illustrated by the linkages provided in Table 2.

From here, it is necessary to identify how to measure TDIs of interest. Measuring team performance is foundational to team science, and there are several resources available for measuring team performance [82]. While there are general best practices for measuring teamwork, such as using observational (rather than self-report) measures, no measure of teamwork is perfect. Instead, researchers should consider the context of their translational team. For example, using team observations to assess SMMs or psychological safety may be preferred when an outside observer is available or when a team is meeting regularly. In this case, observing team meetings with a behaviorally anchored guide for rating team constructs may be an effective and appropriate approach for measuring teamwork. In contrast, when a team is not meeting frequently or where teamwork is less observable, it may be best to evaluate teamwork with brief surveys of members. To develop such surveys, teams can look to psychological research on a construct of interest to find items tapping into each construct.

### Future Directions

This review provides a comprehensive overview of TDIs applicable to translational science teams, providing practical recommendations to enhance effectiveness. However, as translational research continues to target increasingly complex clinical and societal problems, specific issues may arise. The presented interventions are recommendations that can help improve the overall effectiveness of any science team. Additional research understanding the role of targeted interventions is necessary to further enhance science team effectiveness. For example, when implementing targeted team trainings, the learning objectives and overarching goals must be clearly defined prior to implementation [10]. The specific goals for the development of team competencies may vary depending on the research team, thus requiring a deeper understanding of generic translational skills applicable across all research teams compared to teams that meet certain specifications. For instance, research teams that span several geographic boundaries are more likely to meet virtually and place emphasis on a unique set of competencies, like effective writing, that are vital to virtual communication and coordination [83]. Understanding specific characteristics that deviate across various translational teams can significantly guide the development of targeted interventions, consequently further enhancing research effectiveness.

It is also imperative to continue researching the macro-societal conditions that may impact patterns of collaboration. For example, the COVID-19 pandemic has placed increased constraints on various factors known to facilitate science team effectiveness, such as in-person meetings and conferences. Though some translational teams already use virtual methods for communication due to geographic boundaries [84], the level of dependence on virtual methods as well as limited physical proximity imposed on all teams due to the pandemic has yet to be explored in the context of translational science. There is an increased need to understand how translational teams can overcome constraints caused by macro-societal issues such as the COVID-19 pandemic as well as how team interventions may need to be adapted to better facilitate effectiveness in these contexts.

Finally, as CD teams continue to rise in both popularity and size, there is growing evidence that suggests team science functions as a multilevel phenomenon known as a multiteam system (MTS) [85]. A MTS refers to two or more teams that work together interdependently to achieve a superordinate goal [86]. In science teams, component teams are likely to represent collaborative entities (e.g., clinical team, academic university partners, practitioners) that work interdependently to research the defined state of interest. Component team members will need to collaborate both within their own team as well as between other teams. This places a greater emphasis on disciplinary dynamics as well as the structure and dynamics of each component team [87]. Given these complexities, certain skills that are not traditionally as relevant to teams will need to be emphasized to maximize effectiveness, such as boundary spanning and informal interteam leadership [88]. Additional identification of competencies relevant to MTS structures can guide the development of MTS-targeted interventions and benefit CD research MTSs significantly.

### Concluding Thoughts

Given the rise in translational research, alongside advancements in the SciTS literature, several key facilitators to effective team science have been identified. Recognition of these facilitators paved the way for the creation of TDIs (e.g., workshops, training) to grow translational researcher competencies. However, these interventions require extensive time and monetary resources for teams. This integrative review advances the SciTS literature by providing a comprehensive examination of TDIs, highlighting easily implementable solutions to improve effectiveness of translational teams. We reviewed the four stages of TD research alongside key identifiers of each stage so that translational scientists can better diagnose their team’s current development and apply appropriate interventions to enhance overall effectiveness. Additionally, we integrated evidence-based interventions from the groups and teams literature to the context of translational science, including resources for intervention implementation (Table 1) as well as guiding questions (Table 2). In sum, this article provides translational scientists with the necessary tools to overcome barriers that inhibit team science success and therefore enhance team effectiveness.
